# 

**Published:** 1910-06-25

**Authors:** 


**Thomas's Splint.**

Children who have had undoubted hip disease should wear a Thomas's splint for a year. But if the disease tends to progress, if there is increasing pain on slight movement, then a Thomas's splint is of no use; it will be necessary to carry out some surgical measure.—*Mr. Stonham.*

**Extractions and Oral Sepsis.**

In cases of pyorrhœa or oral sepsis always clean the mouth and teeth thoroughly before beginning to extract. This may take a week or longer; if possible, extract by degrees. In every case of oral sepsis there is a great risk of wound infection and toxic absorption after extraction. This applies strongly to debilitated people, especially, perhaps, to tubercular people.—*Mr. J. G. Turner.*

**Arterio-Sclerosis and Iodides.**

No drug will restore elasticity to arteries already stiffened by calcareous deposit, but it is probable that iodides can prevent or delay extension of the sclerosing process. Small doses can be given, 3 or 5 grains, except in syphilitic arteritis. It is supposed that a combination of the iodides of potassium and sodium is more useful, the former being said to have a more powerful resolving action and the latter a more marked hypotensive action. A few drops of tincture of iodine in a claret glass of water before meals has also been recommended, while the newer albuminoid and fatty compounds of iodine have their uses.—*Dr. F. W. Mott.*

THE

### GRADUATES' MEDICAL SCHOOLS.

DIARY FOR THE WEEK, JUNE 27 to JULY 2.

**ROYAL SOCIETY OF MEDICINE,** 15 Cavendish Square, W. (Temporary address during building.)

At 8 p.m.—**June 27, Odontological Section.**
*Discussion* on Mr. Hopewell Smith's paper, "Two odontoceles and some other cysts" (deferred from the April meeting), will be opened by Mr. F. J. Bennett and Mr. W. W. James.
*Casual Communications.*—Mr. Arthur S. Underwood: "Some Egyptian Skulls." Mr. G. Thomson: "A case showing the result of extraction of the six-year-old molars." Valedictory remarks by the President.
5.30.—**June 28, Medical Section.**
*Papers.*—Dr. George Oliver: "A combination of a tactile and auscultatory methods of reading the systolic and diastolic blood-pressure." Dr. William Ewart: "Dorsal percussion of the thorax and of the stomach: and a new stomach sign."

**CENTRAL LONDON THROAT AND EAR HOSPITAL,** Gray's Inn Road, W.C.

At 3.45 p.m.—**June 28,** Mr. J. Gay French, **Tracheoscopy, &c.**
3.45 p.m.—**July 1,** Mr. Chichele Nourse, **External and Middle Ear.**

**MEDICAL GRADUATES' COLLEGE AND POLYCLINIC,** 22 Chenies Street, W.C.

At 4 p.m.—**June 27,** Dr. S. E. Dore, **Clinique (Skin).**
5.15 p.m.—**June 27,** Dr. Theo. Hyslop, **Disorders of Memory.**
4 p.m.—**June 28,** Dr. James Taylor, **Clinique (Medical).**
5.15 p.m.—**June 28,** Dr. R. W. Allen, **The Practical Side of Vaccine Treatment.**
4 p.m.—**June 29,** Mr. Wilfred Trotter, **Clinique (Surgical).**
5.15 p.m.—**June 29,** Dr. E. C. Hort, **Diagnosis and Treatment of Gastric and Duodenal Ulcer.**
4 p.m.—**June 30,** Dr. J. J. Perkins, **Clinique (Medical).**
5.15 p.m.—**June 30,** Mr. Arthur Edmunds, **Intussusception.**
4 p.m.—**July 1,** Mr. E. Kenneth Campbell, **Clinique (Eye).**

**THE POST-GRADUATE COLLEGE,** West London Hospital, Hammersmith, W.

At 10 a.m.—**June 27** and **30, Demonstrations by Surgicsl Registrar.**
10 a.m.—**July 1, Demonstration by Medical Registrar.**
12 noon.—**June 27, Pathological Demonstration.**
11.30 a.m.—**June 28, Demonstrations of Minor Operations.**
12.15 p.m.—Dr. Grainger Stewart, **Practical Medicine.**
5 p.m.—**June 27,** Dr. Pritchard, **Clinical Pathology.**
5 p.m.—**June 28,** Dr. Mansell Moullin, **Gynæcological Cases.**
5 p.m.—**June 29,** Dr. Beddard, **Practical Medicine** (Lecture IV.).
5 p.m.—**July 30,** Mr. Baldwin, **Practical Surgery** (Lecture IV.).
5 p.m.—**July 1,** Dr. Grainger Stewart, **Chronic Muscular Atrophy of Spinal Origin, with Cases.**

**NORTH-EAST LONDON POST-GRADUATE COLLEGE,** Prince of Wales's General Hospital, Tottenham, N.

At 4.30 p.m.—**June 28,** Dr. G. G. Macdonald, **Vaccine Therapy.**
4.30 p.m.—**June 30,** Dr. T. R. Whipham, **The Avenues of Tuberculous Infection.**

**ST. MARK'S HOSPITAL FOR DISEASES OF THE RECTUM,** City Road, E.C.

At 2.30 p.m.—**July 1,** Mr. Aslett Baldwin, **Prolapse and Procidentia.**

**THE BROMPTON HOSPITAL FOR CONSUMPTION AND DISEASES OF THE CHEST,** Fulham Road, S.W.

At 4 p.m.—**June 29,** Dr. Fenton, **Arterio-Sclerosis with some remarks upon Treatment.**

**NATIONAL HOSPITAL FOR THE PARALYSED AND EPILEPTIC,** Queen Square, Bloomsbury, W.C.

At 3.30 p.m.—**June 28,** Dr. H. Tooth, **Clinical Lecture.**
3.30 p.m.—**July 1,** Dr. T. Grainger Stewart, **Cerebral Abscess.**

**LONDON SCHOOL OF CLINICAL MEDICINE,** Seamen's Hospital, Greenwich, S.E.

At 3.15 p.m. — **June 27,** Sir Dyce Duckworth, **Gastric Hæmorrhage.**
3.15 p.m.—**June 30,** Sir W. Bennett.

Lord Strathcona presided at the annual festival dinner of subscribers to the Metropolitan Hospital, Kingsland Road. Among those present were Lord Howard de Walden, chairman of the hospital, Mr. Leopold Rothschild, treasurer, and Sir E. Hay Currie. The subscriptions announced during the evening amounted to £8,794.

### Gifts and Bequests.

Sir John Hollams's estate has been sworn at £601,587 gross, and estate duty of £72,164 has been paid. Sir John has left £100 to each of the following hospitals—namely: St. Thomas's Hospital, the Hospital for Incurables, the Kent Hospital at Maidstone, the Children's Hospital at Cheyne Walk, Chelsea, and the Poplar Hospital for Accidents.

Mrs. F. M. Zarifi and Mrs. L. Lucas have sent £1,000 each for the endowment of beds in response to the special appeal for £10,000 for University College Hospital.

Mr. George Jesson Knight, of Southport, Lancs, has bequeathed £100 each to the Home for Incurables, Manchester, and the Manchester Infirmary.

By the will of the late Mr. George Westerman, of Coalville, the Leicester Infirmary is bequeathed a legacy of £20.

### Jottings.

Metropolitan Hospital Sunday Fund.—The following are among the amounts received at the Mansion House to date: William Herring, Esq., £1,000; Holy Trinity, Chelsea, £460; St. Paul's, Onslow Square, £270; St. John's, Paddington, £261; Messrs. Watts, Watts & Co., Ltd., £210; Bromley (Kent) Parish Church and Missions, £206; F. S. Maton, Esq., £200; F. G. D., £200; Alexander Miller, Esq., £200; Westminster Chapel, £192; Heath Harrison, Esq., £150; Emmanuel, Wimbledon, £147; Aldenham Parish Church, £133; St. Andrew's, Frognal, £130; Brixton Independent Church, £117; City Temple, £113; Temple Church, £108; Annunciation, Chislehurst, £107; A. Dunkelsbuhler, Esq., £105; St. Mark's, Reigate, £103; W. D. Graham Menzies, £100; Lieut.-Colonel Sir Charles Frederick, £100; Central Hill Baptist Church, Norwood, £95; St. Paul's, Westbourne Grove, £93; St. Mary Boltons, £91; Greek Church, Bayswater, £89; St. Anne's, Wandsworth, £78; St. Magnus the Martyr, London Bridge, £70; St. James's, West Hampstead, £67; St. Mary, Shortlands, £67; Beckenham Church and Mission, £62; Christ Church, Newgate Street, £54; Weybridge Parish Church, £53; St. John's, Redhill, £51; Hampton Court Palace, £51; G. P. Gooch, Esq., £52; Messrs. J. H. Vavasseur & Co., Ltd., £52; Mrs. Gooch, £50; Gray's Inn Chapel, £50; Mrs. C. E. Layton, £50; Sir John Ramsden, £50; Mrs. Kenneth M. Clark, £50; Farm Street Roman Catholic Church, £50; Norman McCorquodale, Esq., £50; Russell D. Walker, Esq., £50; Mrs. Brook, £50; St. John's, Putney, £49; Guards' Chapel, Wellington Barracks, £45; St. James's, Garlickhithe, £43; Christ Church, Victoria Street, £44; St. Peter's, Walton, £41; All Saints', Fulham, £40; St. Michael's, Betchworth, £40; St. Albans, Streatham, £40; St. John's, Blackheath, £38; St. Matthew's, Brixton, £35; Bromley (Kent) Wesleyan, £35; Chapel Royal, St. James's, £39; St. John's, Downshire Hill, £38; St. Philip's, Sydenham, £34; St. Anselm's, Hatch End, £34; West Wickham Parish Church, £33; St. John's, Old Malden, £33; Francis Reckitt, Esq., £32; Parish Church and Chapel of Ease, Ilford, £32; St. Luke's, West Norwood, £31; Epsom Parish Church, £31; St. Matthew's, Westminster, £31; St. Peter's, Clapham, £31; C. W. Drabble, Esq., £30; St. Mary's, Primrose Hill, £30; Christ Church, Greenwich, £29; St. Stephen's, Hampstead, £29; Orpington Churches, £28; St. Mary the Virgin, Putney, £27; John S. Gilliat, Esq., £26; St. John's, Sidcup, £26; St. Peter's, Dulwich Common, £26; Keston Parish Church, £26; St. Mary (Old Church), Stoke Newington, £26; St. Thomas, Brentwood, £26; Miss Gabriel, £25; St. Giles-in-the-Fields, £25; Merton Parish Church, £25; Septimus Vaughan Morgan, Esq., £25; Baron E. B. d'Erlanger, £25; Harold Russell, Esq., £25; Otto Beit, Esq., £25; H. Dent Brocklehurst, Esq., £25; St. Matthew's, West Kensington, £25; St. Anne's, Highgate Rise, £24; St. Mary's, Graham Street, £24; Effra Road Church, Brixton, £24; St. Jude's, Brixton, £24; St. John's, Plumstead, £23; St. Augustine's, South Kensington, £22; St. Cyprian's, Brockley, £22; St. Mary Magdalene, Enfield, £22; Jackson's Lane Wesleyan, Highgate, £22; St. James's, Lambeth, £21; Holy Trinity, South Wimbledon, £21; Theydon Bois Parish Church, £21; Farnborough Parish Church, £21; Southwark Cathedral, £21; "In Memoriam," Crosby Lockwood (S.L. and D.E.L.), £20; St. Dionis, Parsons Green, Fulham, £15 3s. 1d. Total, about £21,000.

We understand that about £21,000 has been received by the Lord Mayor at the Mansion House as the result of the annual collections on June 12 in aid of the Hospital Sunday Fund.

An Irish terrier at a Putney hotel has collected no less than £30 for the West London Hospital, Hammersmith, and other charities, by accosting visitors and thrusting his money-box on their attention. His efforts were due to his owner's gratitude for what the hospital had done for one of his employés on whom a dangerous operation had been performed successfully.

King Edward's Hospital Fund has received ten guineas from the "children and staff of the National Refuges, in lieu of a memorial wreath to King Edward." Queen Alexandra has written through the Hon. Sidney Greville to the secretaries of the National Refuges for Homeless and Destitute Children, 164 Shaftesbury Avenue, saying that she is deeply touched by this kind thought, and approving of the way in which the gift has been made.

The Guild of the New Hospital for Women is arranging to hold a sale of "useful and fancy" articles on November 17, 18, and 19, with the object of defraying the cost of the Guild cot for 1910 and for the expenses of the Convalescent Cottage, Aldbury, for the next two years. Contributions will be gratefully received by any of the Sale Committee, of which Miss Hamilton, M.D., 11 Brunswick Square, and Miss Bastow, School of Medicine for Women, are members.

The jubilee of the Seaside Convalescent Hospital, Seaford, was celebrated by an "At Home" at the hospital last Saturday. Mr. Goslett delivered an address on the work of the hospital, and said that 31,000 patients had passed through the institution during the fifty years of its existence, and that only twelve of them had died at the hospital. Only this week the King has granted his patronage to the institution.

On Wednesday, June 15, a "Pound Day" was held in the Board Room of Worcester General Infirmary, when the Mayoress (the Hon. Mrs. A. P. Allsopp) presided. Numerous gifts, from a pound upwards, of every kind of grocery, fruit, vegetables, and drapery were offered, some coming from former patients of the institution. The wards were thrown open, and during the afternoon many visitors inspected them.

### To Hospital Secretaries and Others.

We invite contributions from any of our readers, and shall be glad to pay, according to length, for items of news for the institutional section (including appointments, resignations, gifts, etc.) which we may publish. All payments for manuscripts used are made as early as possible after the beginning of each quarter.

### COMING EVENTS OF THE WEEK.

Sunday, June 26.—Visiting Day at the Hospitals.
Meeting in support of Jewish Hospital, Pavilion Theatre, Mile End, E., 4.30 p.m.
Monday, June 27.—Carnival at Stanningley in aid of the Leeds Hospital.
Thursday, June 30.—Royal Caledonian Asylum Annual Dinner, Hôtel Métropole, 7.
Friday, July 1.—Guy's Hospital Biennial Dinner, Connaught Rooms, 7.30.
Saturday, July 2.—League of Mercy, Lambeth-Kennington District. Mrs. Hornby Steer "At Home," 52 Avenue Road, Regent's Park, 4 p.m.
Dartford District. Mrs. Carl Jay "At Home," Blendon Hall, Bexley, 3.30 p.m.

### APPOINTMENTS.

Ivor Back, Esq., M.B., F.R.C.S., has been appointed assistant surgeon to St. George's Hospital in the place of Lawrence Jones, Esq., M.D., F.R.C.S., resigned.

Mr. J. K. Jameson, M.B., C.B., has been appointed to the chair of anatomy at Leeds University, vacant by the resignation of Professor Griffith, who has accepted the professorship of medicine.

Mr. E. V. Oulton, M.B., B.C. Cambridge, M.R.C.S., L.R.C.P., has been appointed an assistant inspector of the first class and surgeon to the Ophthalmic Hospitals of Egypt.

The Council of the Royal Free Hospital School of Medicine for Women has appointed Mr. Percy Flemming, M.D., F.R.C.S., to be lecturer in ophthalmology, and Mrs. Vaughan-Sawyer, M.D., B.S., to be lecturer in gynæcology.

Dr. C. J. Coleman has been appointed Medical Officer of Health to the Lincoln City Council.

Dr. Scurfield, the Medical Officer of Health for Sheffield, has been nominated as examiner in preventive medicine and public health at the University of Oxford for three years, from October 10 next.

Mr. Alfred Kaye Jarrett, M.B., C.M., L.R.C.P., L.R.C.S. Edin., L.F.P. & S. Glas., has been appointed Medical Officer of Health to the Bridlington Corporation and medical superintendent of the borough sanatorium.

Dr. Wolferstan Thomas, assistant lecturer in the Liverpool School of Tropical Medicine, has been appointed to the directorship of the new laboratories at Manaos in the State of Amazonas, Brazil. He has also been commissioned to proceed to Porto Velho, on the Madeira river, to investigate diseases endemic to that district which tend to retard the commercial progress of the country.

Mr. Cecil A. Joll has been appointed second house surgeon to the Leicester Infirmary. Mr. Joll took his B.Sc. Lond. in 1905; L.D.S. Eng. 1907; M.R.C.S. L.R.C.P., April 1909; M.B., B.S. Lond., F.R.C.S. Eng. 1910. He has had a distinguished career at Bristol University, University College, the London, and other hospitals. He held resident posts at the Bristol General Hospital and the London Hospital. He gained highest place in honours in the M.B., B.S. examination, and the University gold medal with distinction in surgery, medicine, pathology and forensic medicine, and public health.

Mr. A. D. Child, M.B., Ch.B. Edin., has been appointed assistant house surgeon to the Leicester Infirmary. He was formerly assistant house physician at the Nottingham General Hospital.

**The Institution Library.**

"Burdett's Hospitals and Charities," 1910. (Net, post free.) ... ... ... ... ... ... ...10s. 11d.
"Hospital Expenditure: The Commissariat." For the use of Managers. ... ... ... ... ... 2s. 6d.
"The District Nursing Association; Hints on how to Start it." (Net, post free.) ... ... ... 1s. 1d.
"A Legal Handbook for the use of Hospital Authorities." ... ... ... ... ... ... ... 2s. 6d.
Ledgers, Charts, Case-papers, and every kind of Institutional Literature.

Of all booksellers or of The Scientific Press, Limited, 28 and 29 Southampton Street, Strand, London, W.C.

THE HOSPITAL. June 25, 1910.

*Name* ..............................................

*Address* ..............................................

..............................................

This Coupon must accompany manuscript or contributions intended for The Hospital.

